# Using supplementary formula of Qing-Hao-Bie-Jia decoction for defervescence of lung cancer-related fever: a literature review and case report

**DOI:** 10.3389/fmed.2026.1717877

**Published:** 2026-02-20

**Authors:** Xiao-Ge Chu, Wei-Heng Zhang, Bin Luo, Feng Yang, Wen-Man Lv, Sheng-Yu Zhou, Zi-Xin Han

**Affiliations:** 1 Hematology Department, Shanghai Beizhan Hospital, Shanghai, China; 2 School of Traditional Chinese Medicine, Beijing University of Chinese Medicine, Beijing, China; 3 Institute of Oncology, Department, Institute of Oncology Shanghai Municipal Hospital of Traditional Chinese Medicine, Shanghai, China; 4 Institute of Information on Traditional Chinese Medicine China Academy of Chinese Medical Sciences, Beijing, China; 5 School of Clinical Medicine, Jiangxi University of Traditional Chinese Medicine, Nanchang, China

**Keywords:** cancer-related fever, case report, lung cancer, Qing-Hao-Bie-Jia decoction, traditional Chinese medicine

## Abstract

**Introduction:**

Cancer-related fever is a common complication of lung cancer that negatively affects patients’ quality of life. Recently, Qing-Hao-Bie-Jia Decoction (QHBJD), as a representative Traditional Chinese Medicine (TCM) formula historically used to treat endogenous heat caused by Yin deficiency, has been increasingly applied in the management of lung cancer-related fever (LCRF). Our investigation endeavored to establish a chain of evidence bridging classical TCM theory and contemporary clinical practice toward the application of QHBJD in treating LCRF as a methodological reference in the real-world setting.

**Methods:**

Our study was made up of three parts: 1) Ancient origins of QHBJD from pre-modern Chinese medical texts; 2) systematic review of QHBJD for treating LCRF from published case reports; 3) a case report treated with QHBJD-derived supplementary formula from our clinical practice.

**Results:**

From our findings, “latent heat in the Yin” is the predominant pathogenesis of cancerous fever among lung cancer patients, who are mainly treated with the therapeutic principle of nourishing Yin and outthrusting heat. In addition, the pathogenesis of included LCRF case reports is complicated, but TCM practitioners can support vital-qi and eliminate pathogenic factors by flexibly adding or subtracting Chinese medicines based on QHBJD for treatment.

**Conclusion:**

In this study, we established a methodological framework merging ancient and modern evidence based on case reports, which prospectively aids future TCM studies in enhancing certainty of the same evidence from empirical medicine to evidence-based medicine.

## 1 Introduction

Cancer-related fever, a common symptom in cancer patients, can significantly affect their survival. It is defined as a non-infectious fever caused either by the cancer itself or by cancer treatment. Current pathophysiological understanding suggests multiple contributing mechanisms: tissue necrosis caused by proliferation of cancerous cells, endogenous pyrogen generated or active substances secreted during metabolic process, cancer-induced secondary pneumonia, and release of necrosis factors owing to necrosis of cancerous cells during anti-cancer therapy (1–5). At present, its conventional management primarily relies on physical cooling and symptom-oriented medication (non-steroidal anti-inflammatory drugs and corticosteroids) during clinical practice of modern medicine, but the clinical challenges that some patients suffer recurrence(s) after drug withdrawal remain notable (5–7). An emerging case report suggests oxybutynin hydrochloride as a potential therapeutic agent, although its safety and effectiveness require validation through prospective clinical trials (8). Since cancer-related fever is also common among patients with lung cancer, more sufferers may benefit from integrated traditional Chinese and modern medicine (TCM-MM) treatment under current limitations of such synthetic drugs (9).

According to theories of traditional Chinese medicine (TCM), cancer-related fever was conceptualized as “febrile illnesses” in ancient times, and it belongs to endogenous fever caused by internal factors. TCM practitioners indicate that in cancer patients with prolonged treatment, it will result in the deficiency of healthy Qi (it is opposite to pathogenic factors, a collective term for physiological functions of the body), consumption of Yin and blood, as well as debilitation of Yang Qi. Thus, some TCM scholars put forward that pathogenesis of cancer-related fever is highly relevant to “Yin fire” (the pathogenic fire produced from improper diet, fatigue, joy, anger, grief, overthinking, Qi, or Yang deficiency), “latent Yang”(a pathological state where Yang becomes trapped or concealed within the body’s interior, failing to disperse properly to the surface), and “cancer-related toxins” (7, 10–12), which promotes precise diagnosis and treatment later. Furthermore, the corresponding strategies of therapeutic formulas were generated: 1) to reduce fever with sweet and warm medicines; 2) to supplement Qi and nourish Yin; 3) to harmonize and release Shao-Yang (13–15), which includes Qing-Hao-Bie-Jia decoction (QHBJD).

QHBJD is one of the common basic prescriptions for patients with cancer-related fever issued by TCM practitioners (16) toward the syndrome of “Yin deficiency leading to internal heat” (YDIH) (a pathological state in which yin fluid fails to control yang and causes a relative hyperactivity of yang and deficiency heat). Meanwhile, guided by the TCM principles of holism and syndrome identification, case reports align well with individualized treatments. Cancer patients often require highly personalized medication, so therapeutic regimens can vary significantly from person to person. Accordingly, we focused on case reports and tried to perform an investigation linking ancient evidence with modern applications on QHBJD for treating lung cancer-related fever (LCRF), which could provide an evidence-based framework to enrich individualization under TCM-MM intervention.

## 2 Materials and methods

There are three sections in our study. First, the term “Qing-Hao-Bie-Jia,” as a keyword, was applied to retrieval among the *Chinese Medical Dictionary*, the Traditional Chinese Medicine Intelligence Center, and other databases, supplemented by manual screening of original texts from ancient TCM books, which served to trace the origins of QHBJD. Second, we performed a systematic review of case reports in modern clinical practice for LCRF using “Qing-Hao-Bie-Jia,” “Cancer-related fever,” and “Lung cancer” as keywords to systematically search among five common databases (viz., CNKI, WanFang, VIP, CBM, PubMed, and Web of Science) from their inception until 21 August 2024. Moreover, the inclusion criteria were only restricted to “P” (LCRF patients), “I” (interventions embracing QHBJD), and “S” (case report), without a control group and constraints on specific outcomes. Although defervescence is the main outcome, secondary outcomes differ from patient to patient, thereby no specific outcome is limited. But the studies lacking a therapeutic outcome would be excluded, and the JBI critical appraisal checklist was used for methodological quality assessment. Since systematic review serves as a tool to preliminarily summarize individualized therapeutic schedules related to QHBJD, with incomplete structured search terms (absence of “C” and “O”), we did not submit a protocol-designed registration. Moreover, the section reported adherence to PRISMA 2020. Third, we reported a LCRF case successfully treated by QHBJD-derived supplementary formula (QHBJD-SF) in our clinical encounters, in accordance with CARE (17, 18) (Supplementary materials) and CARC (Case Report in Chinese Medicine) (19), to share our experience. All data extracted from the literature were presented using qualitative descriptions.

## 3 Origin and ancient application of QHBJD

After removing duplicates and irrelevant entries, a total of 33 records documenting the use of QHBJD were screened out. We then extracted some classic examples (Table 1) (20–26) for elucidating the origins and development of QHBJD.

**Table 1:** Application and examples of QHBJD in ancient TCM books.

Dynasty	Source	Author	Content
Qing	*Item Differentiation of Warm Febrile Diseases* *(Wen-Bing-Tiao-Bian)*	Ju-Tong Wu	When patients feel heat at night and cool in the morning, with sweatless heat retreat, QHBJD could be used (20).
	*Pediatric Medicine Class(Yi-Xue-Ke-Er-Ce)*	Ding-Fen Gao	The sufferers with severely latent heat in the Yin at night are suitable for QHBJD (21).
	*New Theory of Hidden Pathogen(Fu-Xie-Xin-Shu)*	Heng-Rui Liu	Patients with aversion to cold, dizziness, heat from the body, and other symptoms toward latent heat could be treated by QHBJD (23).
	*Guide to Febrile Diseases(Wen-Bing-Zhi-Nan)*	Lou Jie(Compiler)	Fever at night, cold in the morning, and heat retreat without sweat are the symptoms caused by deeply latent heat in the Yin, which means the fever comes from Yin. And QHBJD is adapted for this kind of patient (25).
Late Qing and the early period of the Republic of China	*New Edition of Popular* *Treatise on Febrile Diseases* (Zeng-Ding-Tong-Su -Shang-Han-Lun)	Gen-Chu Yu (Writer); Lian-Chen He (Revise and Enlarge)	Patient suffers from chronic malaria with blood heat due to deficiency of Yin needs to add **POLYGONI MULTIFLORI RADIX** (*Polygonum multiflorum* Thunb.), **MORI FOLIUM** (*Morus alba* L.), **MUME FRUCTUS** (*Prunus mume Sieb*. et Zucc) into QHBJD (22).
	*Reordering Febrile Diseases Interpretation* *(Chong-Ding-Wen-Re-Jing-Jie)*	Lin Shen	A case report was described as “epidemic febrile disease transforming into heat over 10 days after incubation, with mild fever, especially at night, and absence of sweating after defervescence,” and those were indications of applicability to QHBJD (24).
Early period of the Republic of China	*Essentials of Pediatrics* *(Er-Ke-Yao-Lv)*	Ke-Qian Wu	Acute pediatric convulsion with residual evil heat in the blood that patients feel cool in the morning and have fever at night, should be treated with QHBJD (26).

QHBJD was first recorded in the *Item Differentiation of Warm Febrile Diseases* written by Ju-Tong Wu from the Qing Dynasty, and there were two standardized formulas of QHBJD identified in this book. One comprised of **ARTEMISIAE ANNUAE HERBA** (*Artemisia annua* L.), **ANEMARRHENAE RHIZOMA** (*Anemarrhena asphodeloides* Bge.), **TRIONYCIS CARAPAX** *(Trionyx sinensis Wiegmann)*, **REHMANNIAE RADIX** *(Rehjnannia glutinosa Libosch.)*, **MOUTAN CORTEX** *(Paeonia suffruticosa Andr.)*, while the other consisted of **ARTEMISIAE ANNUAE HERBA**, **ANEMARRHENAE RHIZOMA**, **MORI FOLIUM**, **TRIONYCIS CARAPAX, MOUTAN CORTEX,** and **TRICHOSANTHIS RADIX** (*Trichosanthes kirilowii Maxim. or Trichosanthes rosthornii Harms*), which was cited from *A Guide to Clinical Practice with Medical Record*, toward corresponding symptoms of excessive heat caused by malaria that invades meridians of Shao-Yang (20). QHBJD was employed to address “febrile illnesses” with symptoms of morning coolness, nocturnal fever, and defervescence without sweat. Additionally, Wu also described the etiology and mechanism of febrile diseases with YDIH syndrome (20). From then on, subsequent TCM practitioners gradually expanded their application in clinical practice until QHBJD serves as a classical prescription for treating syndromes characterized by night fever, morning coolness, absence of sweating during defervescence, dry lips, dry mouth, and tongue manifestation of red with little fur. Later, TCM followers continued to extend its application to exogenous febrile conditions, such as summer-heat disease, malaria, and other epidemics. For example, a few TCM specialists used QHBJD-SFs for clearing pathogenic heat latent in the liver meridian of foot-Jue-Yin, the gallbladder meridian of foot-Shao-Yang, the kidney meridian of foot-Shao-Yin, the pericardium meridian of hand-Jue-Yin, and the San-Jiao meridian of hand-Shao-Yang (23).

To sum up, a variety of TCM physicians from different dynasties not only expanded QHBJD’s clinical indications, but also extracted the core etiology and pathogenesis applicable to YDIH syndrome, such as “latent heat in the Yin (a pathological state where pathogenic heat is deeply embedded or concealed within the body’s Yin) and “Yin deficiency-induced blood dryness” (the deficiency of yin fluids generates internal heat to consume in blood), which have provided valuable guidance for current application of QHBJD in intervening LCRF.

## 4 Systematic review of LCRF case reports treated by QHBJD

### 4.1 Result of systematic retrieval

**Figure 1:**
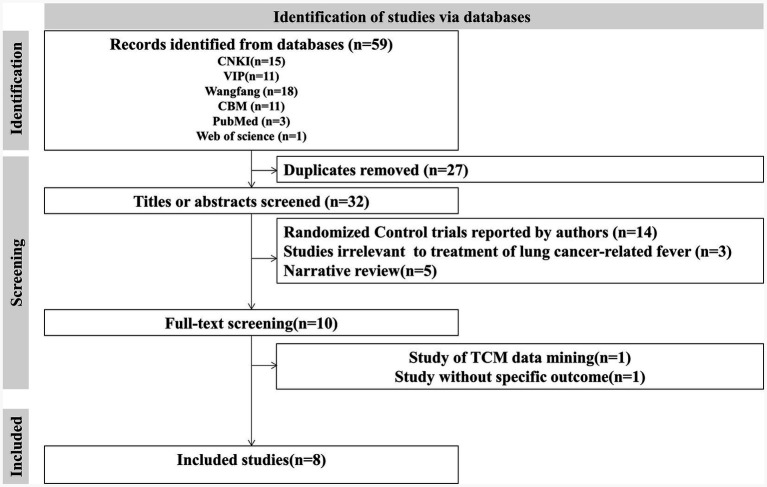
Flow diagram representing the process of identifying eligible studies.

The initial computer-aided retrieval yielded a total of 59 records (Table 2). Subsequently, two authors (SY Zhou and XG Chu) independently screened out the eligible studies that were inspected by a third author (ZX Han). Ultimately, there were eight studies (27–34) included in this study, and the screening process is displayed in Figure 1. Data extraction for the characteristics of included studies (Table 3) was conducted by two other authors (WM Lv and HW Zhang), and the remaining researchers (B Luo and F Yang) resolved inconsistencies between them.

**Table 2:** Strategies of systematic retrieval.

Database	Search strategies {restricted conditions}
CNKI	SU% = Qing-Hao-Bie-Jia AND SU% = Ai-Xing-Fa-Re AND SU% = Fei-Ai
VIP	M = Qing-Hao-Bie-Jia AND M = Ai-Xing-Fa-Re AND M = Fei-Ai
WanFang	Subject: (Qing-Hao-Bie-Jia) and Subject:(Ai-Xing-Fa-Re) and Subject:(Fei-Ai) {Article type: journal research, dissertations, conference papers; Subject terms expanded}
CBM	“Qing-Hao-Bie-Jia” [Common items: Intelligent] AND “Ai-Xing-Fa-Re”[Common items: Intelligent] AND “Fei-Ai” [Common items: Intelligent]
PubMed	QingHaoBiejia [Title/Abstract]
Web of Science	TS = (Qing-Hao-Bie-Jia) OR TI = (Qing-Hao-Bie-Jia) {Preprint Citation Index (Exclude – Database)}

Meanings of the above-mentioned Chinese phonetic alphabets are as follows: (1) Ai-Xing-Fa-Re: cancer-related fever; (2) Fei-Ai: lung cancer.

**Table 3:** Characteristics of included studies.

Source	Gender (Age)	Pathological diagnosis	Treatment	Outcome	Ingredients of QHBJD-SFs
YZ Zhang 2003 (27)	Male (39)	Bronchioloalveolar carcinoma of the lung on the right side	Antibiotics taken for 3 days	Ineffectiveness	**ARTEMISIAE ANNUAE HERBA** 10 g, **TRIONYCIS CARAPAX PRAEPARATA CUM ACETO** 30 g, **REHMANNIAE RADIX** 12 g, **ANEMARRHENAE RHIZOMA** 8 g, **MOUTAN CORTEX** 10 g, **TRICHOSANTHIS RADIX** 10 g, **OPHIOPOGONIS RADIX**^a^ 10 g, **GLYCYRRHIZAE RADIX ET RHIZOMA**^b^ 6 g
			Hormone and Naproxen treated once	Defervescence with recurrence of fever after drug withdrawal	
			10 doses of QHBJD-SF taken	Defervescence without recurrence	
T Zhang 2006 (28)	Female (33)	Bronchiolar squamous cell carcinoma of the lung	Eight doses of QHBJD-SF taken	Defervescence without recurrence	**ARTEMISIAE ANNUAE HERBA** 9 g, **TRIONYCIS CARAPAX PRAEPARATA CUM ACETO** 30 g, **REHMANNIAE RADIX** 20 g, **ANEMARRHENAE RHIZOMA** 9 g, **MOUTAN CORTEX** 12 g, **TRICHOSANTHIS RADIX** 15 g, **OPHIOPOGONIS RADIX** 15 g, **GLYCYRRHIZAE RADIX ET RHIZOMA** 6 g
WW Yang 2012 (30)	Male (62)	Small-cell lung cancer with mediastinal lymph node metastasis	Anti-infective treatment for 7 days	Temperature fluctuated between 37.3 and 37.8°C	**ARTEMISIAE ANNUAE HERBA** 30 g, **TRIONYCIS CARAPAX** 10 g, **ANEMARRHENAE RHIZOMA** 10 g, **LYCII CORTEX** 15 g, **MOUTAN CORTEX** 15 g, **REHMANNIAE RADIX** 30 g, **PAEONIAE RADIX RUBRA**^c^ 15 g, **SOPHORAE FLOS**^**d**^ 15 g, **PHRAGMITIS RHIZOMA** 30 g, **IMPERATAE RHIZOMA**^**e**^ 30 g, **LONICERAE JAPONICAE FLOS**^f^10 g, **GYPSUM FIBROSUM**^g^ 30 g, Liu-Yi-San^#^ 20 g, **SCROPHULARIAE RADIX** 15 g, **OPHIOPOGONIS RADIX** 15 g, **PAEONIAE RADIX ALBA**^h^ 15 g
YY Peng2016 (34)	Male (75)	Lung cancer with local metastasis	Four doses of QHBJD-SF	Defervescence without recurrence	**ARTEMISIAE ANNUAE HERBA** 20 g, **TRIONYCIS CARAPAX** 15 g, **ANEMARRHENAE RHIZOMA** 20 g, **REHMANNIAE RADIX** 15 g, **MOUTAN CORTEX** 15 g, **LYCII CORTEX**^i^ 15 g, **MORI CORTEX**^j^ 10 g, **FORSYTHIAE FRUCTUS**^k^ 10 g, **LONICERAE JAPONICAE FLOS** 15 g, **BAMBUSAE CAULIS IN TAENIAS**^l^ 15 g, **ARISAEMA CUM BILE**^m^ 10 g, **AURANTII FRUCTUS IMMATURUS**^n^ 10 g, **CITRI RETICULATAE PERICARPIUM**^o^ 10 g, **PORIA**^p^ 10 g, **GLYCYRRHIZAE RADIX ET RHIZOMA** 10 g, **FRITILLARIAE THUNBERGII BULBUS**^q^ 10 g
SX Gong 2021 (33)	Male (81)	Lung cancer	Seven doses of QHBJD-SF taken	Defervescence without recurrence	**ARTEMISIAE ANNUAE HERBA** 10 g, **REHMANNIAE RADIX** 10 g, **ANEMARRHENAE RHIZOMA** 10 g, **COICIS SEMEN**^r^ 30 g, **FAGOPYRI DIBOTRYIS RHIZOMA**^s^ 30 g, **TRIONYCIS CARAPAX PRAEPARATA CUM ACETO** 30 g, **SCUTELLARIAE RADIX**^t^ 20 g, **ORYZAE FRUCTUS GERMINATUS**^u^ **TORREFACTIONIS** 15 g, **HORDEI FRUCTUS GERMINATUS TORREFACTIONIS**^v^ 15 g, **RANUNCULI TERNATI RADIX**^w^ 15 g, **CYATHULAE RADIX**^x^ 15 g, **CODONOPSIS RADIX**^y^ 10 g, **ATRACTYLODIS MACROCEPHALAE RHIZOMA TOSTUM CUM MELLE ET FURFURE** 10 g, **PORIA** 10 g, **PINELLIAE RHIZOMA PRAEPARATUM** 10 g, **DIOSCOREAE RHIZOMA TOSTUM** 10 g, **FRITILLARIAE THUNBERGII BULBUS** 10 g, **TRICHOSANTHIS PERICARPIUM**^z^ 10 g, **PLATYCODONIS RADIX**^ab^ 5 g, **GLYCYRRHIZAE RADIX ET RHIZOMA PRAEPARATA CUM MELLE** 5 g
XJ Zhang 2012 (31)	Male (54)	Lung cancer with bony and hepatic metastases	Nine doses of Sheng-Mai-Yin combined with QHBJD-SF	Defervescence	① First diagnosis (3 doses): **PANACIS** **QUINQUEFOLII RADIX**^ac^10 g, **ASTRAGALI RADIX** 30 g, **ARTEMISIAE ANNUAE HERBA** 10 g, **REHMANNIAE RADIX** 24 g, **OPHIOPOGONIS RADIX** 24 g, **DENDROBII CAULIS** 15 g, **GLYCYRRHIZAE RADIX ET RHIZOMA PRAEPARATA CUM MELLE** 10 g, **ANGELICAE SINENSIS RADIX** 15 g, **TRIONYCIS CARAPAX PRAEPARATA CUM ACETO** 3 g, **CYNANCHI ATRATI RADIX ET RHIZOMA**^ad^ 15 g, **OS DRACONIS**^##^ 30 g, **OSTREAE CONCHA**^ae^ 30 g, **CORNI FRUCTUS** 50 g, **IMPERATAE RHIZOMA** 30 g
					② Second diagnosis (6 doses): **PANACIS** **QUINQUEFOLII RADIX** 10 g, **ASTRAGALI RADIX** 50 g, **ARTEMISIAE ANNUAE HERBA** 10 g, **REHMANNIAE RADIX** 24 g, **OPHIOPOGONIS RADIX** 24 g, **DENDROBII CAULIS** 15 g, **GLYCYRRHIZAE RADIX ET RHIZOMA PRAEPARATA CUM MELLE** 10 g, **ANGELICAE SINENSIS RADIX**^af^ 15 g, **TRIONYCIS CARAPAX PRAEPARATA CUM ACETO** 3 g, **CYNANCHI ATRATI RADIX ET RHIZOMA** 15 g, **OS DRACONIS** 30 g, **OSTREAE CONCHA** 30 g, **CORNI FRUCTUS** 50 g, **IMPERATAE RHIZOMA** 30 g, **FORSYTHIAE FRUCTUS** 30 g, **ANEMARRHENAE RHIZOMA** 15 g, **SOLANI CATHAYANI HERBA**^ag^ 30 g, **FAGOPYRI DIBOTRYIS RHIZOMA**^ah^ 50 g, **DIOSCOREAE RHIZOMA**^ai^ 30 g, **LABLAB SEMEN ALBUM**^aj^ 30 g
Qing Xue 2015 (32)	Male (50)	Advanced lung cancer	Anti-infective treatment for 7 days	Ineffectiveness	① First diagnosis (7 doses): **ARTEMISIAE** **ANNUAE HERBA** 10 g, **TRIONYCIS CARAPAX** 30 g, **ANEMARRHENAE RHIZOMA** 10 g, **REHMANNIAE RADIX** 10 g, **MOUTAN CORTEX** 10 g, **LYCII CORTEX** 10 g, **CYNANCHI ATRATI RADIX ET RHIZOMA** 10 g, **CITRI RETICULATAE PERICARPIUM** 10 g, **PORIA** 10 g, **STELLARIAE RADIX**^ak^ 10 g, **PINELLIAE RHIZOMA PRAEPARATUM CUM ZINGIBERE ET ALUMINE**^al^ 10 g, **ALBIZIAE CORTEX**^am^ 10 g, **CURCUMAE RHIZOMA**^an^ 10 g, **GLYCYRRHIZAE RADIX ET RHIZOMA** 5 g
					② Second diagnosis (7 doses): **ARTEMISIAE** **ANNUAE HERBA** 10 g, **TRIONYCIS CARAPAX** 30 g, **ANEMARRHENAE RHIZOMA** 10 g, **REHMANNIAE RADIX** 10 g, **MOUTAN CORTEX** 10 g, **LYCII CORTEX** 10 g, **CYNANCHI ATRATI RADIX ET RHIZOMA** 10 g, **CITRI RETICULATAE PERICARPIUM** 10 g, **PORIA** 10 g, **STELLARIAE RADIX** 10 g, **PINELLIAE RHIZOMA PRAEPARATUM CUM ZINGIBERE ET ALUMINE** 10 g, **ALBIZIAE CORTEX** 10 g, **CURCUMAE RHIZOMA** 10 g, **GLYCYRRHIZAE RADIX ET RHIZOMA** 5 g, **PAEONIAE RADIX RUBRA** 10 g, **GENTIANAE MACROPHYLLAE RADIX** 10 g ③ Third diagnosis (14 doses): **ARTEMISIAE** **ANNUAE HERBA** 10 g, **TRIONYCIS CARAPAX** 30 g, **ANEMARRHENAE RHIZOMA** 10 g, **REHMANNIAE RADIX** 10 g, **MOUTAN CORTEX** 10 g, **LYCII CORTEX** 10 g, **CYNANCHI ATRATI RADIX ET RHIZOMA** 10 g, **CITRI RETICULATAE PERICARPIUM** 10 g, **PORIA** 10 g, **STELLARIAE RADIX** 10 g, **PINELLIAE RHIZOMA PRAEPARATUM CUM ZINGIBERE ET ALUMINE** 10 g, **ALBIZIAE CORTEX** 10 g, **CURCUMAE RHIZOMA** 10 g, **GLYCYRRHIZAE RADIX ET RHIZOMA** 5 g, **MORI RAMULUS**^ao^ 10 g
LY Liu 2012 (29)	Male (49)	Lung cancer with mediastinal metastasis and metastatic hilum of lung	Many kinds of antibiotics taken	Continuous fever and temperature fluctuating between 37.1 and 38.5 °C	**BUPLEURI CHINENSIS RADIX**^ap^ 10 g, **PEUCEDANI RADIX**^aq^ 10 g**, PICRORHIZAE RHIZOME**^ar^10 g, **MUME FRUCTUS**^as^ 4 g, **CYNANCHI ATRATI RADIX ET RHIZOMA** 15 g, **PSEUDOSTELLARIAE RADIX**^at^ 15 g, **ASPARAGI RADIX**^au^10 g, **OPHIOPOGONIS RADIX** 10 g, **ANEMARRHENAE RHIZOMA** 15 g, **EUPHORBIAE HELIOSCOPIAE HERBA**^av^ 15 g, **TRIONYCIS CARAPAX** 15 g, **CORTEX MORI** 15 g, **VESPAE NIDUS** 10 g, **HOUTTYNIAE HERBA**^aw^ 30 g, **LYCII CORTEX** 15 g, **HUMULI SCANDENTIS HERBA**^ax^ 20 g, **FAGOPYRI DIBOTRYDIS RHIZOMA** 15 g
			Decoction of QHBJD-SF combined with Chai-Qian-Mei-Lian-San taken for 14 days	Normal temperature	

^a^*Ophiopogon japonicus* Ker-Gawl.; ^b^*Glycyrrhiza uralen*sis Fisch. or *Glycyrrhiza inflata* Bat.; ^c^*Paeonia ladiflora* Pall. or *Paeonia veitchii* Lynch; ^d^*Sophora japonica* L.; ^e^*Imperata cylindrica Beauv. var. major* C. E. Hubb.; ^f^*Lonicera japonic*a Thunb.; ^g^CaSO_4_•2H_2_O; ^h^*Paeonia lactiflora* Pall.; ^#^It is made up of **GLYCYRRHIZAE RADIX ET RHIZOMA** and **TALCI PUL VIS** (talcum powder); ^i^*Lycium chinense* Mill. or *Lycium barbarum* L.; ^j^(*Morus alba* L.; ^k^*Forsythia suspensa* Thunb. Vahl; ^l^*Bambusa tuldoides* Munro, *Sinocalamus beecheyanus* (Munro) McClure var. *pubescens* P. F. Li or *Phyllostachys nigra* Lodd. Munro var. *henonis* Stapf ex Rendle; ^m^Arisaema erubescens Wall. Schott, Arisaema heterophyllum Bl. or Arisaema amurense Maxim.; ^n^*Citrus aurantium* L. or *Citrus sinensis* Osbeck; ^o^*Citrus reticulata* Blanco; ^p^*Poria cocos* Wolf; ^q^*Fritillaria thunbergii* Miq.; ^r^*Coix lacryma-jobi* L. var. ma-yuen Roman. Stapf; ^s^*Fagopyrum dibotrys* D. Don Hara; ^t^*Scutellaria baicalensis* Georgi; ^u^*Oryza sativa* L.; ^v^*Hordeum vulgare* L.; ^w^*Ranunculus ternatus* Thunb.; ^x^*Cyathula officinalis* Kuan; ^y^*Codonopsis pilosula* Franch. Nannf., *Codonopsis pilosula* Nannf.var.*modesta* L.T.Shen or *Codonopsis tangshen* Oliv.; ^z^*Trichosanthes kirilowii* Maxim. or *Trichosanthes rosthornii* Harms; ^ab^*Platycodon grandiflorum* Jacq. A. DC; ^ac^*Panax quinquefolium* L.; ^ad^*Cynanchum atratum* Bge. or *Cynanchum versicolor* Bge.; ^ae^*Cornus officinalis* Sieb. et Zucc.; ^af^*Angelica sinensis* Diels; ^ag^*Solanum cathayanum* Wu et Huang; ^ah^*Fagopyrum dibotrys* D. Don Har; ^ai^*Dioscorea opposita* Thunb.; ^aj^*Dolichos lablab* L.; ^##^It was derived from either fossilized bones of ancient animals or bones from large modern mammals; ^###^A total of 10 patients suffering from lung cancer were included, and their average age was 54 years. ^ak^Stellaria dichotoma L. var. lanceolata Bge.; ^al^*Pineilia ternata* Thunb. Breit.; ^am^*Albizia julibrissin* Durazz.; ^an^*Curcuma phaeocaulis* Val., *Curcuma kwangsiensis* S. G. Lee et C.F. Liang or *Curcuma wenyujin* Y. H. Chen et C. Ling; ^ao^*Morus alba* L. ^ap^(*Bupleurum chinense* DC. or *Bupleurum scorzonerifolium* Willd.; ^aq^*Peucedanum praeruptorum* Dunn; ^ar^*Picrorhiza scrophulariiflora* Pennell; ^as^*Prunus mume* Sieb. et Zucc.; ^at^*Pseudostellaria heterophylla* Miq. Pax ex Pax et Hoffm.; ^au^*Asparagus cochinchinensis* Lour. Merr.; ^av^*Euphorbia helioscopia* L.; ^aw^*Houttuynia cordata* Thunb.; ^ax^*Humulus scandens* Lour. Merr.

### 4.2 Characteristics of included studies

The eligible studies were published between 2003 and 2021, involved with patients aged 33–81 years with a male-to-female ratio of 7:1. The clinical features documented across eight studies included red face, red tongue, fatigue, limited sweat, dry month, fever (ranging from 37 °C to 39.1 °C in afternoon or at night generally), and taut-thin-and-rapid pulses, which pointed to Yin deficiency secondary to an enduring cancer or/and prolonged chemoradiotherapy. Furthermore, continuous Yin deficiency may disrupt homeostasis, resulting in predisposing the body to pathogenic invasion and accumulating deep-seated pathogenic factors. The TCM syndromes were mainly diagnosed as YDIH and “dual deficiency of Qi and Yin”(a pathological state of simultaneous presence of Qi deficiency and Yin deficiency). Accordingly, the application of QHBJD-SFs focused on nourishing Yin, clearing heat, dispelling pathogenic factors, reinforcing the healthy Qi, and eventually achieving the desired effect of antipyresis. Fortunately, all patients finally obtained defervescence without recurrent fever during the follow-up.

### 4.3 Methodological quality assessment

**Figure 2:**
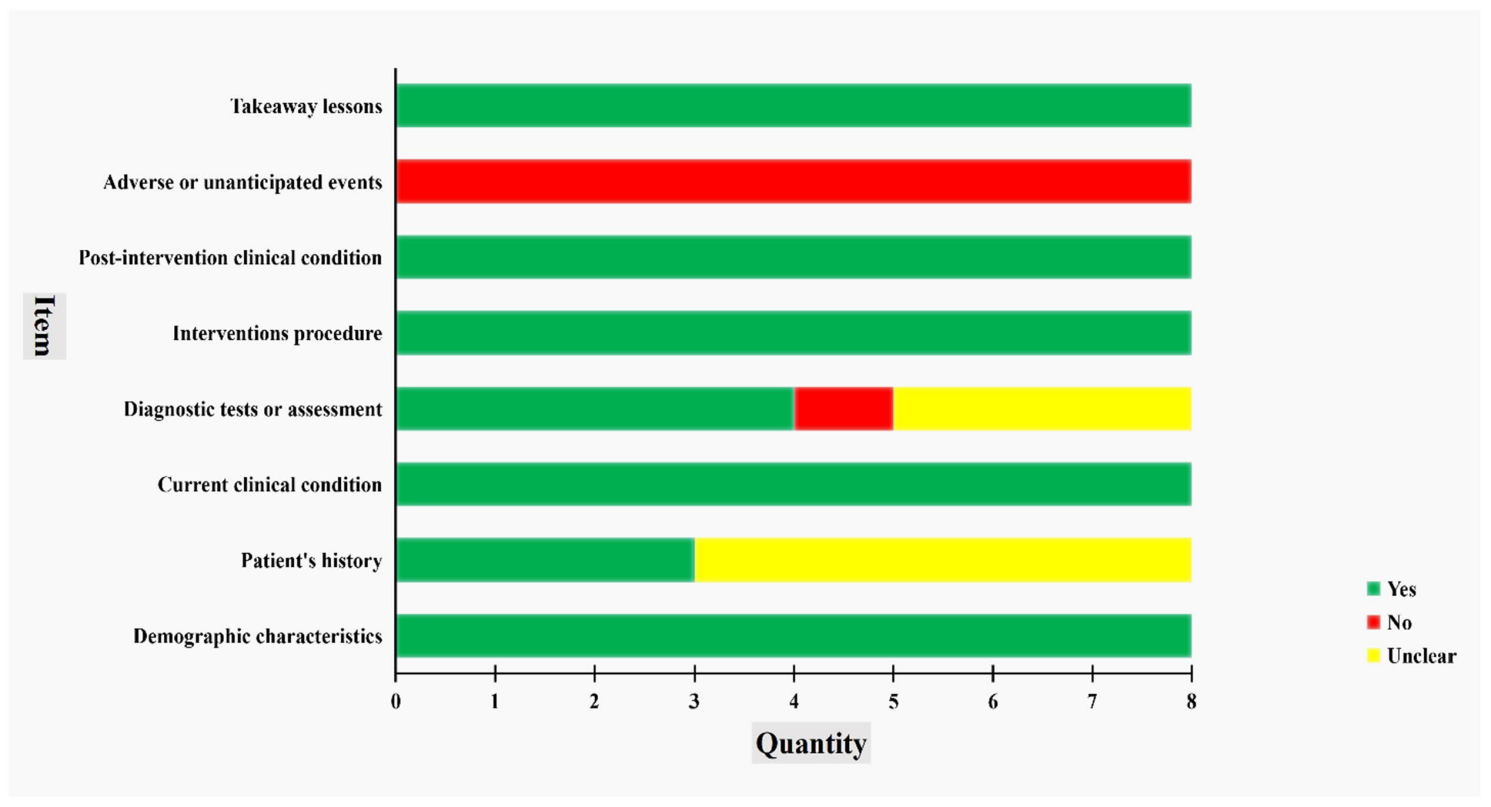
Bias of included case reports.

Thereafter, we executed a critical appraisal of included case reports using the JBI checklist (Figure 2). On one hand, demographic characteristics, current clinical condition, intervention procedures, takeaway lessons, and post-intervention clinical condition were clearly reported in all studies, which indicated strong adherence to documenting patient information, presentation status, treatment details, and prognosis, and deriving clinical implications from the cases. On the other hand, inconsistent reports were shown in the remaining items. First of all, only three studies (37.50%) provided clear descriptions of the patient’s history, while the others were rated as “unclear.” Whereas no timeline graph was found in eight studies. Next, there were four studies (50.0%), three studies (37.50%), and one study (12.50%) to provide clear, unclear, and absent descriptions in diagnostic tests or assessment, respectively, among which only one study (29) displayed detailed photos of TCM syndrome identification. Eventually, none of the adverse or unanticipated events was mentioned.

## 5 LCRF case report treated by QHBJD-SF

### 5.1 Case presentation

A 46-year-old male factory worker with a history of lung adenocarcinoma (T2N0M0, TNM staging of AJCC 8th edition) underwent thoracic resection 2 years before presentation. Postoperatively, he developed persistent low-grade fever lasting 8 months. In May 2021, the patient visited our department and received QHBJD-SF treatment, resulting in the complete resolution of febrile symptoms. At present, his elaborated diagnosis and treatment process was reported as follows. First, common physical examinations were conducted in sequence: 1) temperature was 37.9 °C; 2) pulse was 61 beats per minute; 3) blood pressure was 120/80 mmHg; 4) respiratory rate was 19 breaths per minute; 5) no jaundice was observed on the skin, mucous membranes, or sclera; 6) no significant superficial lymph node was found based on palpation. Second, we inquired about the epidemiological history that he had not been exposed to communicable diseases such as hepatitis, tuberculosis, epidemic areas, contaminated water, blood transfusions, or COVID-19. Third, his medical history was negative for trauma, diabetes, or hypertension. He also denied any history of prior surgical procedures. Fourth, the absence of elevated serum tumor markers, coupled with CT imaging demonstrating expected postoperative changes in the left lung parenchyma, did not suggest any substantial support for recurrence or progression. Fifth, the function of his liver and kidneys was normal. The main results of his biochemical tests are shown in Table 4.

**Table 4:** Patients’ partial blood examination.

No.	Category	Item	Result
1	Complete blood count	C-reactive protein (CRP)	<0.80 mg/L
2		Serum amyloid A (SAA)	5.11 mg/L
3		Red blood cell count	4.58 × 10^12^/L
4		Hemoglobin (Hb)	146 g/L
5		White blood cell count	5.32 × 10^9^/L
6		Neutrophil percentage	52.4%
7		Lymphocyte percentage	32.7%
8		Monocyte percentage	10.0%
9		Eosinophil percentage	4.1%
10		Basophil percentage	0.8%
11		Absolute neutrophil count	2.79 × 10^9^/L
12		Absolute lymphocyte count	1.7410^9^/L
13		Absolute monocyte count	0.53 × 10^9^/L
14		Absolute eosinophil count	0.22 × 10^9^/L
15		Absolute basophil count	0.04 × 10^9^/L
16		Platelet count (PLT)	171 × 10^9^/L
17	Serum tumor markers	Gastrin-releasing peptide precursor (ProGRP)	12.00 U/mL
18		Carbohydrate antigen 50 (CA50)	3.76 IU/mL
19		Carbohydrate antigen 242 (CA242)	7.27 IU/mL
20		Alpha-fetoprotein (AFP)	5.01 ng/mL
21		Carcinoembryonic antigen (CEA)	1.73 ng/mL
22		Carbohydrate antigen 19–9 (CA19-9)	6.22 U/mL
23		Carbohydrate antigen 125 (CA125)	9.00 U/mL
24		Carbohydrate antigen 15–3 (CA15-3)	8.60 U/mL
25		Total prostate-specific antigen (tPSA)	1.090 ng/mL
26		Free prostate-specific antigen (fPSA)	0.438 ng/mL
27		FpSA/tPSA	40
28		Carbohydrate antigen 72–4 (CA72-4)	1.32 U/mL
29		Cytokeratin 19 fragments (CYFRA21-1)	1.85 ng/mL
30		Neuron-specific enolase (NSE)	12.57 ng/mL
31		Ferritin	221.10 ng/mL
32		Squamous cell carcinoma antigen (SCC-Ag)	1.31 ng/mL
33	Biochemistry indexes related to liver function	Alanine aminotransferase (ALT)	27 U/L
34		Aspartate aminotransferase (AST)	22 U/L
35		AST/ALT	0.79↓
36		Lactate dehydrogenase (LDH)	120↓U/L
37		Gamma-glutamyl transferase (GGT)	22 U/L
38		Alkaline phosphatase (ALP)	91 U/L
39		Prealbumin	249 mg/L
40		Cholinesterase	7439.0 U/L
41		Total bilirubin (TBiL)	15.30 umol/L
42		Conjugated bilirubin (CB)	5.6 umol/L
43		Glycocholic acid (GCA)	1.80 ug/ml
44		Glutamate dehydrogenase (GLDH)	6.2 U/L
45		Total bile acids (TBA)	1.9 umol/L
46	Biochemistry indexes related to kidney function	Creatinine (Cr)	62 umol/L
47		Urea	5.78 mmol/L
48		Cystatin C	0.74 mg/L

### 5.2 Diagnostic assessment and clinical findings

To elucidate his etiology, we conducted a TCM syndrome differentiation. The patient was an early-stage lung cancer case who had undergone a 4-week course of chemotherapy following tumor resection. Previously, he had been treated with a modified Xiao-Chai-Hu decoction, targeting Shao-Yang Syndrome through heat-clearing and harmonization, but this failed to relieve his low-grade fever. Currently, his fever (37.8 °C) persists, predominantly occurring in the afternoon. In addition, he often experienced fatigue and wheezing after exercise and had poor sleep at night. His stool and urine remained normal, and his appetite remained routine, with only occasional cough. On examination, his pulse was thin and rapid, accompanied by a light yellow complexion, a dark red tongue, and thin tongue coating. Finally, we considered him as a case of LCRF due to YDIH, judged by absence of bitter and dry month, low-grade fever in the afternoon, his manifestation of tongue and pulse, ruling out infectious etiology (CRP level <0.80 mg/L), as well as inefficacy of previous TCM intervention, which indicated that the patient did not meet the criterion of “Chai-Hu Pattern” (35, 36).

Thus, this case fell falling within the therapeutic scope of QHBJD. Furthermore, supplementing qi and nourishing Yin, clearing heat and removing toxins, softening hardness, and dissipating masses were determined as his therapeutic principles. This case was probably different from typical advanced-cancer cases whose conditions mainly present “local fullness (weighted) and whole emptiness (weightless),” as he had finished his resection over 3 years, where the “local fullness” had been disposed. His intervention should pay attention to the “local emptiness,” in a bid to prevent recurrence, metastasis and developing pre-metastatic microenvironment of the target organs—aligning with the TCM principle of “To address the root cause of long-term disease”—for prolonged survival of cancerous sufferers.

### 5.3 Therapeutic interventions

As a result, we issued a 14-dose prescription of QHBJD-SF that was made up of **TRIONYCIS CARAPAX** 18 g, **ARTEMISIAE ANNUAE HERBA** 15 g, **MOUTAN CORTEX** 9 g, **REHMANNIAE RADIX** 15 g, **ANEMARRHENAE RHIZOMA** 12 g, **ASTRAGALI RADIX** (*Astragalus membranaceus* Bge. var. *mongholicus* Hsiao or *Astragalus membranaceus* Bge.) 30 g, **ATRACTYLODIS MACROCEPHALAE TOSTUM CUM MELLE ET FURFURE RHIZOMA** (*Atractylodes macrocephala* Koidz.) 9 g, **SALVIAE CHINENSIS HERBA** (*Salvia chinensis* Benth.) 30 g, **CREMASTRAE PSEUDOBULBUS PLEIONES PSEUDOBULBUS** 9 g (*Cremastra appendiculata* Makino, *Pleione bulbocodioides* Rolfe or *Pleione yunnanensis* Rolfe), **VESPAE NIDUS** (*Polistes olivaceous*, *Polistes japonicus* Saussure or *Parapolybia* var*ia* Fabricius) 9 g, **PARIDIS RHIZOMA** (*Paris polyphylla* Smith var. *yunnanensis* Hand. -Mazz., or *Paris polyphylla* Smith var. chinensis Hara) 9 g, **GYNOSTEMMAE HERBA** (*Gynostemma pentaphyllum* Thunb. Makino) 15 g, **AKEBIAE FRUCTUS** (*Abebia quinata* Thunb. Decne., *Akebia trifoliata* Thunb. Koidz. or *Akebia trifoliata* Thunb. Koidz. var. *australis* Rehd.) 12 g, **POLYGONATI RHIZOMA PRAEPARATUM CUM VINO FRUMENTI** (*Polygonatum kingianum* Coll. et Hemsl., *Polygonatum sibiricum* Red., or P*olygonatum cyrtonema* Hua) 15 g, **GANODERMA** (*Ganoderma lucidum* Karst. or *Ganoderma sinense* Zhao, Xu et Zhang) 15 g, **EPIMEDII FOLIUM** (*Epimedium brevicomu* Maxim., *Epimedium sagittatum* Maxim., *Epimedium pubescens* Maxim. or Epimedium koreanum Nakai) 9 g, **PHRAGMITIS RHIZOMA** (*Phragmites communis* Trin.) 30 g, **SCROPHULARIAE RADIX** (*Scrophularia ningpoensis* Hemsl.) 15 g, **OSTREAE CONCHA** (*Ostrea gigas* Thunberg, *Ostrea talienwhanensis* Crosse or *Ostrea rivularis* Gould) 30 g, **PRUNELLAE SPICA** (*Prunella vulgaris* L.) 12 g, **CITRI RETICULATAE SEMEN** (*Citrus reticulata* Blanco) 6 g, **GALLI GIGERII ENDOTHELIUM CORNEUM** (*Gallus domesticus* Brisson) 12 g, and **GLYCYRRHIZAE RADIX ET RHIZOMA** (*Glycyrrhiza uralensis* Fisch., *Glycyrrhiza inflata* Bat., or *Glycyrrhiza glabra* L.) 9 g.

### 5.4 Outcomes and follow-up

**Figure 3:**
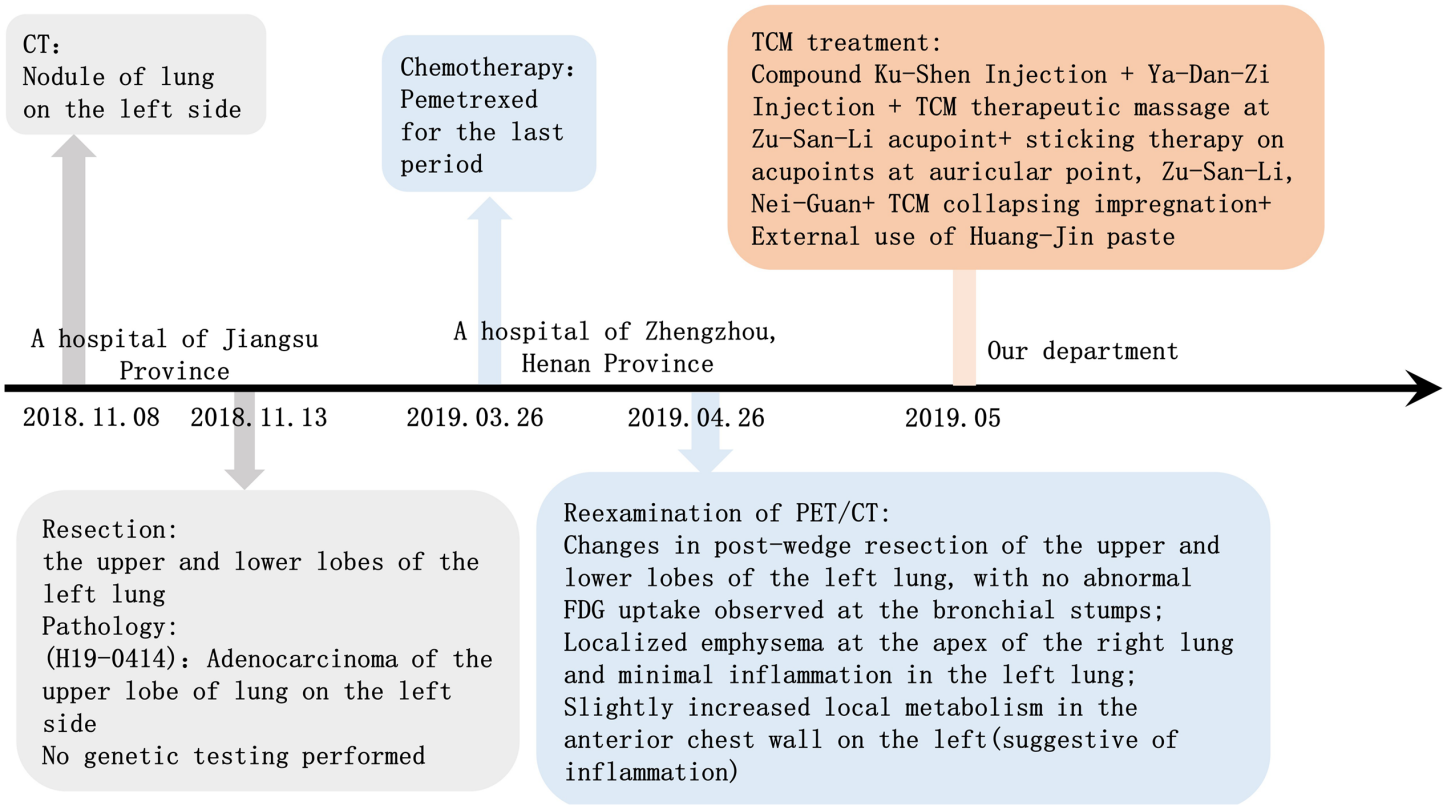
Timeline of the case report before accepting treatment of QHBJD-SF.

The patients exhibited good adherence to the medication, without any adverse events detected during the course of the treatment. Finally, his symptom of low-grade fever disappeared and did not recur during the follow-up until 1 August 2025. His former diagnosis and treatment details are shown in Figure 3.

## 6 Discussion

### 6.1 Summary

We conducted a three-step investigation to generate new insight into ancient and modern evidence of QHBJD for LCRF based on case reports. From our findings, the characteristics of low-grade fever in our case shared major clinical features with those in the included studies (Table 3), which met the TCM diagnostic criteria of YDIH that corresponded to classical descriptions of “latent heat in the Yin” in ancient TCM books.

### 6.2 Practical implications

Notably, our case developed steadily during his long-term anti-cancer treatment, while continuous low-grade fever did impact his quality of life. After excluding infectious fever based on CRP level, QHBJD was chosen to nourish Yin and outthrust latent heat (37). In our opinion, his lung function was impaired by long-term depletion of Yin and Qi due to prolonged disease course. In line with TCM theories, insufficient Qi of the lung not only caused fatigue and weakness but also resulted in most body fluid accumulating in his lungs, which eventually induced YDIH. Afterward, another 18 Chinese medicines were added to QHBJD, for reinforcing healthy Qi to eliminate pathogenic factors. Concretely, **ASTRAGALI RADIX** and **ATRACTYLODIS MACROCEPHALAE TOSTUM CUM MELLE ET FURFURE RHIZOMA** can supplement and strengthen the spleen; **SALVIAE CHINENSIS HERBA, CREMASTRAE PSEUDOBULBUS PLEIONES PSEUDOBULBUS**, **VESPAE NIDUS, PARIDIS RHIZOMA, OSTREAE CONCHA, CITRI RETICULATAE SEMEN**, and **GYNOSTEMMAE HERBA** can clear heat, remove toxins, soften hardness and dissipate masses; **AKEBIAE FRUCTUS** and **PRUNELLAE SPICA** can regulate Qi and soften hardness; **POLYGONATI RHIZOMA PRAEPARATUM CUM VINO FRUMENTI, GANODERMA, EPIMEDII FOLIUM, PHRAGMITIS RHIZOMA,** and **SCROPHULARIAE RADIX** can nourish Yin and tonify Yang of kidney; **TRIONYCIS CARAPAX, ARTEMISIAE ANNUAE HERBA, MOUTAN CORTEX, REHMANNIAE RADIX** and **ANEMARRHENAE RHIZOMA** can nourish Yin and clear heat. Briefly, our QHBJD-SF synergistically addressed Yin nourishment, heat clearing, mass dissolution, and Qi supplement in order to protect the body against “latent heat in the Yin.”

### 6.3 Potential mechanisms of QHBJD for LCRF

Recent investigators demonstrate that QHBJD can effectively improve basic metabolism and enhance the non-specific immune level of the body, thereby regulating the overall human system to relieve low-grade fever, whose mechanism is promisingly associated with upregulating CD15 and downregulating CD13, CD14, CD33, and CD34 (38). In addition, modern pharmacological researches have revealed that QHBJD can reduce the extent of macrophage infiltration in tumor tissues, potentially by intervening in the COX/LOX pathway to block the metabolism of arachidonic acid, and its toxicity is weaker than that of the inhibitors targeting the COX-2/5-LOX pathway (39). Moreover, some network pharmacology findings revealed a series of putative mechanisms, such as bioactive constituents (quercetin, kaempferol, luteolin, and icariin) of QHBJD to target spots therapeutic points (e.g., VEGFA, CASPS, AKT1, MMP9, TNF, and IL6) and to modulate the signaling pathways of TNF, IL-17, PI3K-Akt (40), as well as 141 potential biological processes with adjusting inflammation level and improving cancer-related fever through several signal pathway of TNF, MAPK NOD-like receptor, TOLL-like receptor, FOXO (41). Furthermore, the following core components exert direct antitumor effects: artemisinin and its derivatives (42), polysaccharides of **TRIONYCIS CARAPAX,** peptides and bone collagen, catalpol and stachyose of **REHMANNIAE RADIX,** saponin A-III of **ANEMARRHENAE RHIZOMA**. A and paeonol have shown their effect on inducing apoptosis in cancer cells to a certain extent, as well as on inhibiting tumor proliferation, growth, and metastasis significantly.

### 6.4 Research gaps and limitations

We found a severe gap in safety reporting and history presentation toward current LCRF cases treated by QHBJD, where adverse drug reactions/events were not mentioned and timeline graphs were not provided. Hence, it is not comprehensive enough for a timeline graph that is inconvenient for readers to capture core information.

However, there were certain limitations in this study. For one thing, given the limited techniques for compilation and digitization of ancient TCM literature currently, our study might not display an all-embracing and comprehensive ancient evidence of QHBJD, even though we carefully collated and extracted the included original texts from ancient books to guarantee their validity and precision. For another, although the evidence generated from this study could be a theoretical support for clinical encounters, case reports are graded as level-V evidence of TCM studies (43) and are also classified as evidence of very low-certainty in the GRADE system (44). In a word, the above-mentioned modern evidence primarily serves as a direct descriptive evidence in the real-world setting, without a control group and long-term follow-up, which has economical and operable advantages (45, 46), but also needs more prospective studies with rigorously designed protocols to complement our findings.
