# Supplementary material for: Using supplementary formula of Qing-Hao-Bie-Jia decoction for defervescence of lung cancer-related fever: a literature review and case report
Source: Front Med (Lausanne). 2026 Feb 20;13:1717877. doi: 10.3389/fmed.2026.1717877 (PMC12963053; doi:10.3389/fmed.2026.1717877)
Supplement: Supplementary file 1 [file Supplementary_file_1.pdf]

Reporting checklist for case report or case series

Based on the CARE guidelines.

Instructions to authors

Complete this checklist by entering the page numbers from your manuscript where readers will find each of the items listed below.

Your article may not currently address all the items on the checklist. Please modify your text to include the missing information. If you are certain that an item does not apply, please write "n/a" and provide a short explanation.

Upload your completed checklist as an extra file when you submit to a journal.

In your methods section, say that you used the CAREreporting guidelines, and cite them as:

Gagnier JJ, Kienle G, Altman DG, Moher D, Sox H, Riley D; the CARE Group. The CARE Guidelines: Consensus-based Clinical Case Reporting Guideline Development

		Reporting Item	Section
Title			
	#1	The area of focus and “case report” should appear in the title	Title
Keywords			
	#2	Two to five key words that identify topics in this case report	keywords
Abstract			
Introduction	#3a	What is unique and why is it important?	1 Introduction
	#3b	The patient’s main concerns and important clinical findings.	5.1 Case presentation; 5.2 Diagnostic assessment and clinical findings
	#3c	The main diagnoses, interventions, and outcomes.	5.2 Diagnostic assessment and clinical findings; 5.3 Therapeutic interventions; 5.4 Outcomes and follow-up;
Conclusion	#3d	What are one or more “take-away” lessons?	6.2 Practical implications
Introduction			
	#4	Briefly summarize why this case is unique with medical literature references.	6.1 Summary
Patient information			
	#5a	De-identified demographic and other patient information.	5.1 Case presentation

		Reporting Item	Section
	#5b	Main concerns and symptoms of the patient.	5.1 Case presentation
	#5c	Medical, family, and psychosocial history including genetic information.	5.1 Case presentation
	#5d	Relevant past interventions and their outcomes.	5.2 Diagnostic assessment and clinical findings
Clinical findings			
	#6	Relevant physical examination (PE) and other clinical findings.	5.1 Case presentation; 5.2 Diagnostic assessment and clinical findings
Timeline			
	#7	Relevant data from this episode of care organized as a timeline (figure or table).	Figure 3
Diagnostic assessment			
	#8a	Diagnostic methods (PE, laboratory testing, imaging, surveys).	5.1 Case presentation; 5.2 Diagnostic assessment and clinical findings
	#8b	Diagnostic challenges.	5.2 Diagnostic assessment and clinical findings 6.2 Practical implications
	#8c	Diagnostic reasoning including differential diagnosis	5.2 Diagnostic assessment and clinical findings 6.2 Practical implications
	#8d	Prognostic characteristics when applicable	n/a
Therapeutic Intervention			
	#9a	Types of intervention (pharmacologic, surgical, preventive).	5.3 Therapeutic interventions
	#9b	Administration of intervention (dosage, strength, duration)	5.3 Therapeutic interventions
	#9c	Changes in the interventions with explanations.	n/a
Follow up and outcomes			
	#10a	Clinician and patient-assessed outcomes when appropriate	n/a
	#10b	Important follow-up diagnostic and other test results.	n/a
	#10c	Intervention adherence and tolerability (how was this assessed)?	n/a
	#10d	Adverse and unanticipated events.	n/a
Discussion			
	#11a	Strengths and limitations in your approach to this case.	6.2 Practical implications 6.4 Research gaps and limitations

		Reporting Item	Section
	#11b	Discussion of the relevant medical literature.	6.3 Potential mechanisms of QHBJD for LCRF
	#11c	The rationale for your conclusions.	6 Discussion
	#11d	The primary “take-away” lessons from this case report.	6.2 Practical implications
Patient perspective			
	#12	The patient can share their perspective on their case	n/a
Informed consent			
	#13	The patient should give informed consent.	Supplementary materials

None The CARE checklist is distributed under the terms of the Creative Commons Attribution License CC-BY-NC. This checklist can be completed online using https://www.goodreports.org/, a tool made by the EQUATOR Network in collaboration with Penelope.ai

Written Informed Consent

Patient Informed Items

- The author of the submitted article (complete list: Xiao-Ge Chu, Wei-Heng Zhang, Bin Luo, Feng Yang, Wen-Man Lv, Sheng-Yu Zhou, Zi-Xin Han) has provided me with a detailed description of the manuscript whose content will be published in the journal named *Frontiers in medicine*, and the journal will not use the information for commercial purposes such as advertisement or packaging.
- I understand that my privacy is adequately protected in the article and information relating to identifiable text will not be disclosed.
- This paper does not include my personal images.
- The case report is published worldwide and the journal is primarily intended for academic communication in healthcare field, but its information may also be accessible to the public, including journalists, who are not physicians.
- I am able to withdraw my consent at any time prior to publication. Once this informed consent has been delivered for publication, this consent cannot be withdrawn.
- I consent to the publication of my above-mentioned information in *Traditional Medicine Research* as well as its relevant media and professional databases.

Signature of patient: ____________________

Signature of attending doctor: ____________________

Date: 2025.6.24
